# Foot and mouth disease outbreak investigation and estimation of its economic impact in selected districts in northwest Ethiopia

**DOI:** 10.1002/vms3.208

**Published:** 2019-11-11

**Authors:** Belege Tadesse, Amanuel Tesfahun, Wassie Molla, Eyasu Demisse, Wudu T. Jemberu

**Affiliations:** ^1^ College of Veterinary Medicine and Animal Sciences Department of Veterinary Epidemiology and Public Health University of Gondar Gondar Ethiopia; ^2^ School of Veterinary Medicine Wollo University Dessie Ethiopia; ^3^ Gondar Zuria District Livestock Resource office Gondar Ethiopia

**Keywords:** economic impact, Ethiopia, foot and mouth disease, morbidity, outbreak

## Abstract

Foot and mouth disease (FMD), a highly contagious and economically important disease of cloven‐hoofed animals, is endemic in Ethiopia. Foot and mouth disease outbreak investigation and follow‐up studies were undertaken to identify the causative serotype, determine the morbidity and mortality, and estimate the economic impact of the outbreaks in selected districts of Northwest Ethiopia. The serotype of FMD virus involved in the outbreaks was identified by antigen detection ELISA from clinical samples. Morbidity, mortality and economic impact of the outbreaks were assessed based on data collected from 738 smallholder farmers in a mixed crop‐livestock (MCL) production system and from five dairy farms in the commercial dairy production system. The outbreaks were confirmed to be due to FMD virus serotype O. The animal level morbidity in clinically affected cattle herds was 68.1% for MCL production system and 54.5% for commercial dairy farms. The mortality in cattle in the MCL system was 0.4% and no mortality was recorded in the commercial dairy farms. The animal level morbidity in sheep and goats in the infected flocks was 35.7% but no mortality was seen in these species. The herd/flock level morbidity of FMD in outbreak affected kebeles of MCL system was 57.2% for cattle and 8% for sheep and goats. The economic losses due to milk loss, draught power loss, mortality and treatment cost were on average USD 34 (interquartile range: 9.4–44.4) per affected herd in the MCL system and this was statistically significantly lower than the USD 459.1 (interquartile range: 400.0–486.2) per affected farm in the commercial dairy farms (*p* < .05). These economic losses have significant impact in the livelihood and income of affected farmers in both production systems. Future work should focus on the implementation of control measures that mitigate the economic impact of the disease*.*

## Graphical abstract

This study followed up selected outbreaks of FMD that occurred in northwest Ethiopia. Based on the detail follow‐up of the cases, the serotype of the causal virus was identified, the morbidity and mortality of FMD in different species of ruminants and different types of production system were determined, and the economic losses to farmers due to the outbreaks were estimated.

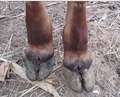

## 1 INTRODUCTION

Foot and mouth disease (FMD) is a contagious transboundary and economically devastating viral disease of cloven‐hoofed animals including both domestic and wildlife species (Mahy, 2005; Thomson, Vosloo, & Bastos, 2003). The disease is caused by foot and mouth disease virus (FMDV) that is classified within the genus *Aphtovirus* and family *Picornaviridae* and consists of seven different serotypes (A, O, C, Asia1, SAT (South African territories)1, SAT2 and SAT3) with many subtypes (OIE, 2017). It is characterized by vesicular eruptions in the oral cavity, foot and udder, these lesions being associated with fever, lameness, salivation and anorexia (Grubman & Baxt, 2004).

Foot and mouth disease is considered as the most important livestock disease in the world in terms of its economic impact (James & Rushton, 2002). The economic impact of FMD in endemic areas can be separated into direct and indirect losses (Knight‐Jones & Rushton, 2013). The annual economic impact of FMD in terms of visible production losses and vaccination costs in endemic regions of the world is estimated between USD 6.5 and 21 billion, while outbreaks in FMD‐free countries and zones cause losses of more than USD 1.5 billion per year (Knight‐Jones & Rushton, 2013). Few case outbreak studies that were conducted in different parts of the world reported a significant impact of FMD in the smallholder settings (Jemberu, Mourits, Woldehanna, & Hogeveen, 2014; Rast, Windsor, & Khounsy, 2010; Young, Suon, Andrews, Henry, & Windsor, 2013).

Foot and mouth disease virus is endemic in Ethiopia in all production systems and a large number of outbreaks were reported every year (Ayelet, Gelaye, Negussie, & Asmare, 2012; Jemebru et al., 2016). Based on data over the years 2007–2012, annual district level incidence of FMD outbreak was estimated at 0.24, 0.39 and 0.85 per district year in the crop livestock mixed, pastoral and market‐oriented districts, respectively, and the outbreaks were caused by serotypes O, A, SAT 2 and SAT 1 (Jemberu et al., 2016). Serological studies of FMD undertaken in different parts of the country reported seroprevalence ranging from 5.6% to 24.2% (Desissa, Tura, Mamo, & Rufae, 2014; Jenbere, Etana, & Negussie, 2011; Mekonen, Beyene, Rufael, Feyisa, & Abunna, 2011; Mesfine, Nigatu, Belayneh, & Jemberu, 2019; Mohamoud, Tessema, & Degefu, 2011; Zerabruk, Romha, & Rufael, 2014).

Despite the occurrence of several outbreaks in the country, only very few outbreaks are investigated for their economic impact and confirmed by laboratory diagnosis. Proper outbreak investigation helps to identify the prevalent serotype of the virus circulating and to assess the economic impact of the outbreaks. The current study was undertaken to investigate causal serotypes, record the morbidity and mortality and estimate economic impact of FMD outbreaks that occurred in 2017–2018 in selected areas in northwest Ethiopia.

## 2 MATERIALS AND METHODS

### 2.1 The study area and study population

**Figure 1:**
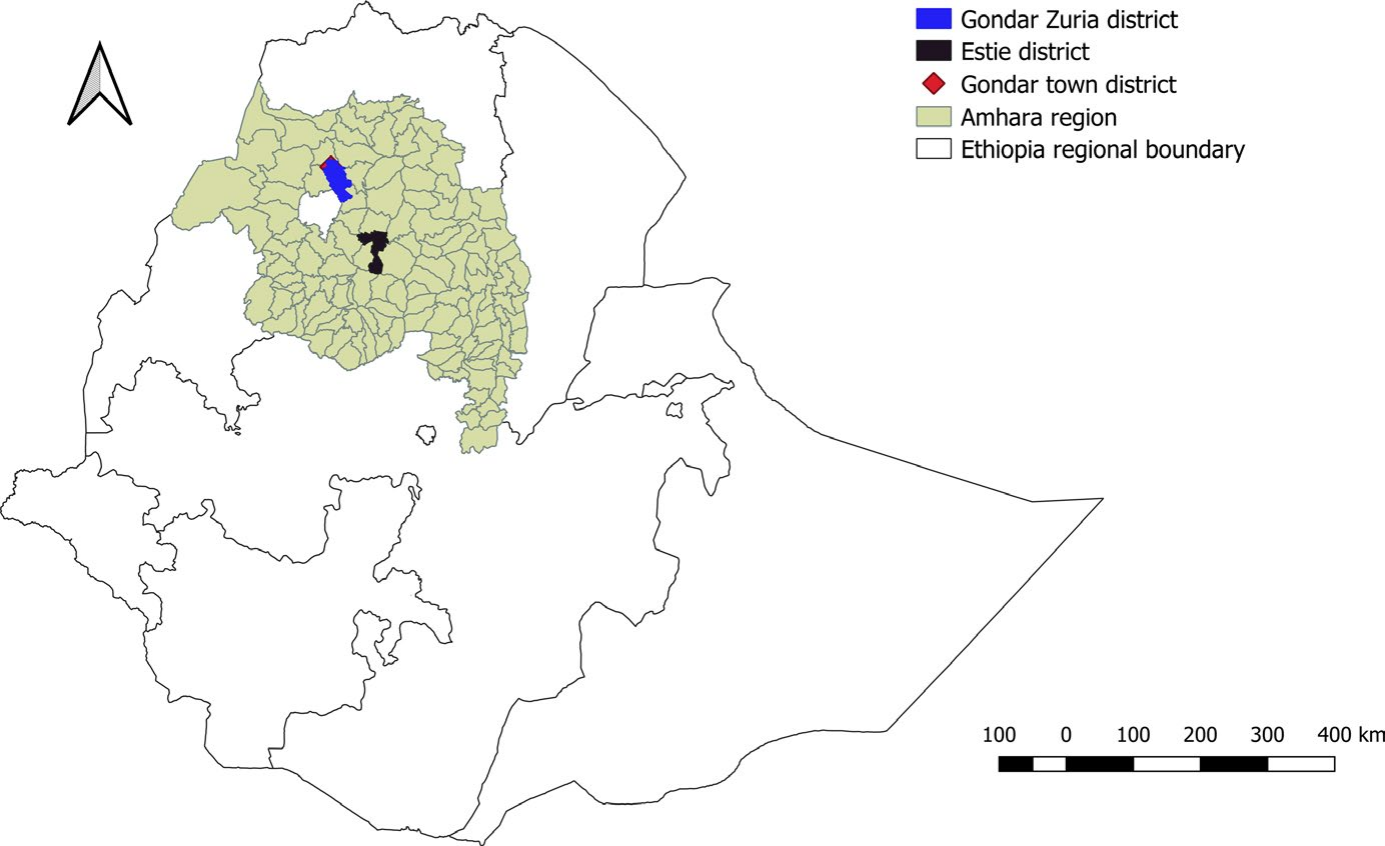
Map of  Ethiopia showing Amhara region and the study districts

The study was conducted in three districts: Estie, Gondar Zuria and Gondar town districts in Amhara region of Ethiopia (Figure 1). The districts were selected for the presence of active outbreaks during the study period, September 2017 to February 2018. Animals in Estie and Gondar Zuria districts were managed in a mixed crop‐livestock (MCL) production system where cattle were primarily kept on smallholdings to provide draught power for crop production and milk for household consumption. Whereas cattle in Gondar town district were managed under intensive commercial dairy production system where cattle were primarily kept for production of milk for market.

The study populations include all household herds (that include cattle, sheep and goat owned by individual households) in the MCL system and dairy farms in the commercial production system found in outbreak affected kebeles (subunits of district) of the study districts.

### 2.2 Outbreak confirmation and serotype identification

From all outbreak affected kebeles, 16 clinically FMD suspected animals with active lesions (blisters or recently ruptured vesicular lesion in the oral cavity and interdigital space) were sampled for viral detection and serotype identification. About 1 g of epithelial tissue samples was collected aseptically from each animal with the help of tissue forceps. The epithelial tissue samples were placed in a sampling bottle containing phosphate‐buffered saline (PBS) solution. The samples were labelled with species, sex, and age of the animals and type of tissue and place of origin, and placed in the ice box containing ice, transported to the University of Gondar veterinary microbiology laboratory and stored at +4°C. After a few days, the collected tissue samples were shipped in an ice‐cooled container to the National Animal Health Diagnostic and Investigation Center, Sebeta, Ethiopia for testing.

The samples were tested using sandwich ELISA (IZSLER, Brescia, Italy) aimed at detection of the virus and identifying of viral serotypes involved in the infection. Sandwich‐ELISA was performed with selected combinations of anti‐FMDV monoclonal antibodies (MAb), used as coated and conjugated antibodies. The sandwich ELISA kit was designed for detection and typing of FMDV type O, A, C, SAT1 and SAT2. A pan‐FMDV test, detecting any isolates of O, A, C, Asia1 and SAT serotypes, was also included in the kit to complement the specific typing. The test was performed based on the manufacturer's recommendation and OIE (2017).

Briefly, the test was performed as follows: The tissue samples were grinded by pestle and mortar by adding sterile sugar and one millilitre of PBS, centrifuged and the supernatant were taken for processing. About 25 μl of dilute buffer was dispensed into all wells of the test plate, then 25 μl of previously diluted samples using ELISA buffer and ready‐to‐use controls (negative and positive controls) was dispensed into the appropriate wells of the test plate pre‐coated with recombinant FMD viral antibody. The plates were sealed using the enclosed plate sealer and incubated for 1 hr at room temperature (20–25°C). After incubation, micro‐plates were filled with about 200 μl of washing solution and the incubated plate was washed three times in 3 min interval and taped by towel. Then, 50 μl of conjugate A (row A to F) and conjugate B (raw G to H) was dispensed to each well, respectively. Incubation repeated once again for 1 hr at room temperature (20–25°C) and then after the incubation period, the plate was washed for four times as above and taped with towel. Then, 50 μl chromogen or substrate solution was dispensed into each well and incubated in dark for 20 min at room temperature. Next, 50 μl of stop solution was added to all wells, and the colour change was appreciated by necked eye. Finally, the results were read using a spectrophotometer at 450‐nm wavelength to obtain the optical density (OD) and the result was interpreted according to the kit protocol.

### 2.3 Recording of morbidity and mortality

In the outbreak affected kebeles and commercial farms, the total number of herds and animals were first registered and then a follow‐up was made regularly to record morbidity and mortality. Follow‐up was made weekly until the outbreak was ended. During follow‐up, animals were observed from distance for evidence of salivation and lameness and when animals show sign of fever and depression they are caught and examined for lesion. Owners were also asked for any symptoms of FMD in their animals during the interim period.

In each outbreak affected kebele, the number of animals at risk, age and sex category of animals (cattle, sheep and goat), number affected and number died due to FMD were recorded to determine morbidity and mortality at animal and herd levels. The animal level morbidity was determined as the number of clinically affected animals divided by the total number of animals at risk. Animal level morbidity was determined both for clinically affected herds in which case the animals at risk are all animals in the clinically affected herds, and for all monitored herds in which case the animals at risk are animals in all monitored herds (all herds in outbreak affected kebeles). The herd level morbidity was determined as the number of positive herds (herds with one or more clinically affected animals) divided by the total number of herds. Mortality was determined as the number of animals died of FMD divided by the total number of animals at risk.

### 2.4 Collecting economic impact data from the outbreaks

For estimating the economic impact of the outbreaks, production loss and disease control data were collected from the farmers using a questionnaire. For this purpose, a structured questionnaire was adapted from Jemberu et al. (2014) (Appendix). All the 743 herd owners (390 from Estie and 348 from Gondar Zuria districts) from the MCL production system in the outbreak affected kebeles and five outbreak affected commercial dairy farms from Gondar town were enrolled in the study. The questionnaire was administered by face‐to‐face interview immediately at the end of the outbreak (when occurrence of new cases stopped) in each herd/farm. Before the interview, an oral consent was obtained from each participant herd owner after being informed on the purpose of the study, the risks and benefits of participation in the study and the right of refusing to participate in the study if they do not want to.

The questionnaire was designed primarily to record morbidity and mortality, production losses and treatment expenditures in different categories of cattle. Herd owners were asked to estimate the daily milk production before and during illness, duration of illness for lactating cows, the milk price per litre, the duration of illness for working oxen that caused loss of draught power, the renting price of an ox, the price of treatment per animal, labour time spent for the extra care and treatment of affected animals and daily wage for manual labour. All prices were based on local markets. Cattle were grouped into six different categories based upon their production status at the time of the outbreak. These categories were (1) lactating cows, (2) pregnant cows; cows that are pregnant and not giving milk, (3) dry cows; cows that are neither pregnant nor giving milk, (4) draught oxen; all adult male animals that are used for plowing, (5) young stock; both male and female animals older than 1 year and not inseminated or used for plowing and (6) calves; young cattle up to 1 year of age. Financial information was collected first in Ethiopian currency (Birr) and later converted to USD at an exchange rate of 27 Birr = USD 1.

### 2.5 Estimation of economic losses

The economic impact of FMD both in the MCL and commercial production systems was determined by an estimation of the direct visible production losses such as milk loss, draught power loss and mortality loss and treatment costs by adapting the method described by Jemberu et al. (2014). Economic loss calculations were done at herd/farm level and the average loss from individual clinically affected animals was also estimated.

#### 2.5.1 Milk loss

Economic losses due to milk loss per FMD affected herd were calculated as:

$$L{\text{milk}}_{i}=N{\text{cow}}_{i}\times Q_{i}\times T{\text{milk}}_{i}\times P\text{milk,}$$

where *L*milk*_i_* represents the economic losses due to milk loss for herd *i*; *N*cow*_i_* the number of lactating cows affected in herd *i*; *Q_i_* the average quantity of milk lost in litres per affected cow per day in herd *i*; Tmilki the average duration of illness in days of affected lactating cows in herd *i*, *P*milk the price of milk per litre. In the MCL production system, an average price of 15 birr per litre was used based on the milk price obtained by the market‐oriented farmers in the surrounding urban centres (Mekaneyesus and Maksegnit town). It was also 15 birr per litre for commercial dairy farms in Gondar town. Commercial farms and some of the MCL herd owners estimated the volume of the daily milk produced in litres. However, the majority of MCL herd owners estimated the volume of milk produced by each FMD‐affected cow using the local container called ‘Gucho’ or bucket which normally is used for milking. This was later converted to litre after filling the container with water to the level indicated by the owner and measured using a graduated jug to know the amount of milk lost per day during the outbreak.

#### 2.5.2 Draught power loss

Economic losses due to draught power loss per herd was calculated as:

$$L{\text{draft}}_{i}=N{\text{oxen}}_{i}\times \left(T{\text{draft}}_{i}\times \;\text{adj}\right)\times P\text{draft,}$$

where Ldraft_i_ represents the economic losses due to draft power loss for herd *i*; *N*oxen*_i_* the number of oxen affected in herd *i*, *T*draft*_i_* the average duration of illness in days of an affected ox in herd *i*, adj an adjustment factor and Pdraft the price of draft power rent of an ox per day. Draught power for crop production (plowing and threshing) is not needed throughout the year because of seasonality in crop production. Moreover, not all days in the planting season are effective working days. According to Goe (1987), draft oxen in smallholder farms of Ethiopia work only for about 65 days per year. The probability that a day on which an ox is ill coincides with an effective working day equals, therefore, to 65/365 (0.178). This ratio was used as an adjustment factor (adj) to change the days of illness to actual working days lost.

#### 2.5.3 Mortality loss

The mortality loss was set equal to the market value of the animal that died. Thus, the economic loss due to mortality per herd was calculated by considering the six different categories of animals that died and their corresponding market price as:

$$L{\text{mort}}_{i}=\sum_{\left(j=1\right)}^{6}N\text{MC}ij\times PCj,$$

where *L*mort*_i_* represents the economic losses due to FMD induced mortality in herd *i*; *N*MC*_ij_* is the number of animals that died in each category *j* of herd *i*, *P*C*j* is the average price of animals in category *j*.

#### 2.5.4 Treatment cost

Foot and mouth disease control costs are considered to consist of vaccination, diagnosis and medication costs and extra labour costs for seeking treatment for sick animals. There was no vaccination or any preventive measure in the study areas and cost of control were mainly related to antibiotic treatment of infected animals for bacterial complication. So the cost of FMD treatment was calculated as:

$${\text{TrCost}}_{i}=\left(N{\text{Tr}}_{i}\times P{\text{Tr}}_{i}\right)+\left(N\text{hours}L_{i}\times P\text{dl}\right),$$

where TrCost*_i_* represents the treatment cost for affected herd *i*; *N*Tr*_i_* the number of animals treated in herd *i*; *P*Tr*_i_* the average per head expenditure for FMD treatment in herd *i*; *N*hoursL*_i_* the average number of working hours lost by the attendant/owner of herd i for seeking treatment for sick animals, and *P*dl the average payment rate of a replacement labour (manual labour) per hour in that locality.

#### 2.5.5 Overall economic losses

The overall economic losses per individual herd were aggregated as the sum of all losses arising from milk loss, draught power loss, mortality loss and treatment costs:

$${\text{TEL}}_{i}={\text{Milk}}_{i}+{\text{Draft}}_{i}+L{\text{Mort}}_{i}+{\text{TrCost}}_{i},$$

where TEL*_i_* represents the total economic losses for herd *i*, Milk*_i_* the economic losses due to milk loss in herd *i*, Draft*_i_* the economic losses due to draught power loss for Sherd *i*; and Mort_i_ the economic losses due to mortality in herd *i* and TrCost*_i_* represents the treatment costs for affected herd *i*.

### 2.6 Data management and statistical analysis

The collected data were entered into Excel spread sheet (Microsoft Corp. Washington, USA) and the data were checked for errors of entry. The economic loss models were programmed in the excel spread sheet. The data were further imported to Stata version 14.0 (Stata Corp, Texas, USA) for statistical analysis. Statistical analyses were conducted to test the significance of differences in morbidity and mortality and economic losses between production systems. A proportion test was used to evaluate the differences in morbidity and mortality between production systems. An independent sample *t* test was used to evaluate differences in herd level economic losses between production systems and between districts within the MCL production system. A p value less than 0.05 was considered as significant in the statistical analyses.

## 3 RESULTS

### 3.1 Outbreak confirmation and serotype identified

Out of 16 tissue samples from 16 animals tested for the presence of FMD viral antigens by sandwich ELISA test, 13 samples (81.25%) were positive for FMDV. All of the eight samples taken from Estie district were positive for FMDV, whereas only four out of six samples taken from Gondar Zuria district and one out of two samples taken from Gondar town district were positive for FMDV. All 13 positive samples were identified to be due to FMD serotype O.

### 3.2 Morbidity and mortality

In all, 13 out of 40 and eight out of 36 kebeles were affected by the FMD outbreak in Estie and Gondar Zuria districts, respectively. In the FMD outbreak affected kebeles of the MCL districts, 738 (57.2%) of the herds were affected by the outbreak (Table 1).

**Table 1:** Foot and mouth disease morbidity at kebele and herd level in the MCL system

District	No. of kebeles	Kebeles affected (%)	No. of herds monitored	No. of cattle in the herds	Herds affected (%)
Estie	36	8 (22.2)	745	7,164	390 (52.3)
Gondar Zuria	40	13 (32.5)	546	6,683	348 (63.7)
Overall	76	21 (27.63)	1,291	13,847	738 (57.2)

The animal level morbidity in the clinically affected MCL cattle herds and commercial dairy farms was 68.1% (95% CI: 66.7–69.3) and 54.5% (95% CI: 43.5–65.2), respectively. The animal level morbidity difference between the two production system was statistically significant (*p* < .05) (Table 2). The animal level morbidity of cattle in all monitored MCL herds was 24.4% (3385/13847). In cattle, the mortality at animal level in the affected herds was 0.4% (95% CI: 0.24–0.62) and case fatality was 0.58%. No mortality was reported in the commercial dairy farms (Table 2).

**Table 2:** Foot and mouth disease morbidity and mortality in affected herds of MCL system and commercial dairy farms

Production system	District	No. of affected herds/	No. of cattle in affected herds	No. of FMD positive cattle	Morbidity (95%CI)	No. of cattle dead	Mortality (95% CI)
MCL	Estie	390	2,423	1,634	67.4 (65.5**–**69.3)	6	0.25 (0.09**–**0.47
	Gondar Zuria	348	2,551	1,751	68.6 (66.6**–**70.4)	14	0.55 (0.30**–**0.91)
	Overall	738	4,974	3,385	68.1 (66.7**–**69.3)	20	0.4 (0.24**–**0.62)
Commercial dairy	Gondar town	5	88	48	54.5 (43.5**–**65.2)	0	0

Note

*p* = .007 for proportion test of differences in morbidity between animals in MCL production system and commercial dairy farms.

In sheep and goat flocks, the flock level morbidity was 8.2%. The animal level morbidity in affected flocks and in all monitored flocks was 35.7% and 3.0%, respectively (Table 3). No mortality was recorded in sheep and goat flocks.

**Table 3:** Foot and mouth disease outbreak morbidity and mortality in sheep and goat flocks in the MCL system

District	No. of flocks monitored	No sheep and goats in the monitored flocks	No. of infected flocks (%)	No of Sheep and goats in infected flocks	No. of infected sheep and goats in monitored flocks (%)	No. of infected sheep and goat in infected flocks (%)	No. of sheep and goat died (%)
Estie	416	1,542	48(11.5)	192	63 (4.1)	63 (32.8)	0
Gondar Zuria	352	1,507	15(4.3)	60	27 (1.8)	27 (45)	0
Overall	768	3,049	63(8.2)	252	90 (3.0)	90 (35.7)	0

### 3.3 Economic losses of foot and mouth disease outbreaks

The overall economic losses due to FMD were aggregated from its impact on mortality, milk reduction, draught power loss and treatment cost. Only cattle were included for estimating economic losses from the outbreak.

The mean economical loss per dead animal was estimated as USD 194.45. The loss varied from USD 30.8 in the young stock to USD 388.9 in draught ox, which simply reflects the market price of these category of animals. There was no mortality loss in commercial dairy farms.

The daily milk yield reduction due to FMD was on average by 51.7% for a period of about 25.3 days. The mean daily milk loss per FMD‐affected lactating cow was 1.85 litre (L). Breed wise, it was 1.4 L in local cows and 2.9 L in cross breed cows. The mean economic losses due to milk loss per affected milking cow were USD 26, USD 22.3 in the MCL production system and USD 97.5 in the commercial dairy farms (Table 4).

**Table 4:** Economic losses due to milk loss per affected lactating cow by breed, production system and district

Production system		District	Daily milk yield (L) before the illness	Daily milk loss due to FMD(L)	Duration of reduced production (days)	Quantity of milk lost (L)	Economic losses (USD)^a^
			Mean(range)	Mean(range)	Mean(range)	Mean(range)	Mean(range)
MCL		Estie	3.32 (1.5–10)	1.65 (0.5–4)	26 (10–90)	42.9(5–360)	23.8 (2.8–200)
		Gondar Zuria	2.7 (1.7–7)	1.6 (0.5–4)	23.7 (7–80)	37.92 (3.5–320)	21.1 (1.9–177.8)
		Overall	3 (1.5–10)	1.62 (0.5–4)	24.8 (7–90)	40.2 (3.5–360)	22.3 (1.9–200)
Commercial		Gondar town	12.35 (9–15)	5.35 (3.5–6)	32.8 (25–65)	175.5 (87.5–390)	97.5 (48.6–216.7)
Overall			3.6 (1.5–15)	1.85 (0.5–6)	25.3 (7–170)	46.8 (3.5–270)	26 (1.9–200)
economic loss by breed	Local		2.5 (1.5–10)	1.4 (0–4)	24.5 (7–80)	34.3 (0–320)	19.1 (0–179.2)
	cross		5.9 (3–15)	2.9 (1–6)	26.6 (12–90)	77.14 (12–540)	42.9 (6.7–302.4)

a The currency exchange during the current study period was assumed as USD1 = 27 birr.

The mean number of effective working days lost per affected ox was 4.8 days (1.4–16 days) resulting in mean loss of USD 13.51 per affected ox (Table 5).

**Table 5:** Economic losses due to draught power loss per affected ox in the MCL system

District	No. of infected oxen	Duration of FMD illness (days)	Effective working days lost	Economic loss per ox (USD)^a^
		Mean (range)	Mean (range)	Mean (range)
Estie	462	25.3 (8**–**60)	4.5 (1.4**–**10.7)	12.51 (3.89**–**29.75)
Gondar Zuria	480	28.7 (8**–**90)	5.1 (1.4**–**16)	14.18 (3.89**–**44.48)
Overall	942	27 (8–90)	4.8 (1.4–16)	13.34 (3.89–44.48)

a 2.78 USD rent price of draft power per day was used

Only 2.2% of FMD‐affected cattle got treatment for secondary bacterial complication. The mean diagnosis and medication cost per treated animal was USD 1.91. It varied between districts and it was USD 1.5 in Gondar Zuria district, USD 2 in Estie district and USD 8 in Gondar town. The mean herd level treatment costs were USD 0.16 in the MCL and USD 5.7 in the commercial herds.

Based on the individual animal losses documented in the preceding paragraphs, the herd level economic loss was estimated and presented in Table 6. The mean overall economic loss (mortality loss, milk loss, draft loss and treatment cost) per infected herd was USD 34 (interquartile range (IQR): 9.4–44.4) in the MCL production system and USD 459.1 (IQR: 400.0–486.2) in the commercial dairy farms (Table 6). The mean overall herd level economic losses in the MCL production system were significantly lower than in the commercial dairy farms (*p* < .05). There was a significant difference in mean overall economic loss among districts of the MCL production system (*p* < .05). In the MCL production system, at infected herd level, the largest component of the economic loss was due to draught power loss (USD 17.2) followed by milk loss (USD 13.9). Whereas, in commercial dairy farms, the largest mean economic loss was due to milk loss (USD 453.4). FMD treatment cost was the least contributor to herd level losses in the MCL herds, while in commercial dairy farms the least loss was due to mortality.

**Table 6:** Mean overall economic losses of FMD per affected herd by district and production system in USD

Production system	District/farm	Milk losses	Mortality losses	Draught power losses	Treatment cost	Overall economic losses
		Mean (IQR)	Mean (IQR)	Mean (IQR)	Mean (IQR)	Mean (IQR)
MCL	Estie	10.8 (0,13.3)	2.9 (0,0)	15.2 (1.9,23.5)	0.28 (0,0)	29.2 (9.4,34.8)
	Gondar Zuria	17.4 (0, 22.2)	2.52 (0,0)	19.4 (5.3, 30.7)	0.02 (0,0)	44.02 (9.8, 51.9)
	MCL Overall	13.9 (0, 16.6)	2.73 (0, 0)	17.2 (4.7, 26.7)	0.16 (0,0)	34 (9.4, 44.4)
Commercial	Gondar district	453.4 (400, 474)	0 (0, 0)	0 (0, 0)	5.7 (0, 10.2)	459.1 (400, 486.2)

Note

*p* = .0,007 for differences in mean overall herd level economic loss between districts within the MCL production system. *p* = .0001 for differences in mean overall herd level economic loss between the MCL production system and commercial dairy farms.

IQR, Interquartile range

The mean economic losses per infected animal were USD 7.4 and USD 47.83 in the MCL production system and in the commercial dairy farms, respectively. The mean economic losses per animal in infected MCL herds and commercial dairy farms were USD 5.5 and USD 26, respectively.

## 4 DISCUSSION

In this study, FMD outbreaks that occurred in northwest Amhara region were closely investigated through a longitudinal follow‐up of cases until the outbreaks stopped. As such, the study produced detailed and reliable data on the morbidity, mortality and associated economic impacts.

The outbreaks in all districts were confirmed by detecting antigen of FMDV in 81% of the clinical samples submitted for laboratory diagnosis and the FMDVs detected were all serotype O. Serotype O used to be the most frequently identified serotype of FMDV in Amhara region and also in Ethiopia in general (Ayelet et al., 2009; Jemberu et al., 2016). In Amhara region, serotype O and A are the commonly isolated serotypes, serotype O being more dominant (Menda, Jenberie, Negusssie, & Ayelet, 2014; Negussie, Kyule, Yami, Ayelet, & Jenberie, 2011).

The 57.2% herd level and 68.1% animal level morbidity in the current study in the MCL production system are lower than the 85% herd level and 74% animal level morbidity reported by Jemberu et al. (2014) in the same type of production system. The lower herd level morbidity might be due to limited movement of animals during the current outbreak period. The current outbreak occurred during the cropping season in which mixing of herds from different kebeles is minimal and this might reduce the herd level morbidity. The background immunity from previous outbreaks (no vaccination was practiced in both areas) may be also a factor for this difference. A high animal level morbidity ranging between 60% and 100% was also reported previously in the MCL production system in eastern parts of Ethiopia (Mersie, Tafesse, Getahun, & Teklu, 1992; Roeder, Abraham, Mebratu, & Kitching, 1994) and in smallholders in other countries like a morbidity of 74% in Cambodia (Young et al., 2013).

The animal level morbidity in the current study is higher than the 19.6% morbidity reported by Negussie et al. (2011) in Ethiopia. The 54.5% morbidity in the commercial dairy farms in the current study was lower than the 64.23% reported in central Ethiopia from commercial dairy farms ( (Beyi, 2012), 62% in a large dairy farm in Kenya (Lyons et al., 2015) and 100% overall morbidity in naive animals in Egypt (Abed El‐Rahman et al., 2006). This variation in morbidity may be due to the difference in breed of animals, strain of the virus circulating in these different outbreaks and also difference in background immunity due to previous outbreak or vaccination. In the current study, the animal level morbidity in the infected herd was higher in the MCL production system (68.1%) than in the commercial farms (54.5%). The animals in the commercial farms were crossbreds and this lower morbidity was unexpected as improved breeds of cattle are more susceptible (Kitching, 2002; OIE, 2013). However, it was observed that in commercial dairy farms infected animals were isolated until they recover and this might contribute to lower transmission within herds as compared with the MCL system where this practice was absent. It should be also noted that the relatively small number of farms included from the commercial dairy system in the study may affect the morbidity comparison.

The mortality rates of 0.4% in the MCL production system in cattle in the current study were lower than the finding of Jemberu et al. (2014) (2.4%) in North Wollo, whereas it is higher than the 0.12% mortality rate reported in Ethiopia (Negussie et al., 2011). The mortality observed in this study is much lower than reports from other counties, for example, the 7.3% mortality reported in Cambodia (Young et al., 2013). There was no mortality in the commercial dairy farms in the current study, while a mortality of 4.01% was reported in central Ethiopia in commercial dairy farms (Beyi, 2012). In an outbreak of FMD in a large commercial dairy farm in Kenya, similarly, very low mortality rate of 0.4% was reported (Lyons et al., 2015). This wide difference in mortality could be due to differences in age composition of herds as the disease's mortality is known to be higher in young calves (OIE, 2013) and could also be due to factors mentioned above for morbidity differences.

The 35.7% animal level morbidity of sheep and goats in the infected herds reported in this study was higher than the morbidity of 4.8% reported in Bangladesh (Chowdhury, Rahman, Rahman, & Rahman, 1993) in sheep and goats. Given the mild nature of the clinical signs of FMD‐infected sheep and goats that can be easily missed (OIE, 2013), the current observed morbidity can be considered high indicating that the disease could be clinically important for small ruminants as well.

In the current study, the average duration of milk loss was 25.3 days, which was 24.8 days in the MCL production system and 32.8 days in commercial dairy farms. This is greater than the reports from central Ethiopia (Beyi, 2012) and Eastern part of Ethiopia (Mersie et al., 1992), where milk loss of at least for 19 and 20 days was complained by farmers, respectively. Another study in Ethiopia reported an average duration of milk loss for 33 days in the MCL production system (Jemberu et al., 2014), which agrees with the finding of the current study in the commercial dairy farms, but higher than in the MCL production system. The average duration of milk loss in the current study was close to the report from Bangladesh (Chowdhury et al., 1993), where the period of illness for FMD‐affected cattle varied from 16 to 26 days (average 22.7 days). However, the milk drop may continue for several months after the outbreak and some cows may totally not return to normal production after clinical recovery. For instance, there was a 3.9% milk drop after clinical recovery in dairy farms in Kenya (Mulei, Wabacha, & Mbithi, 2001).

The mean milk loss per day per cow due to FMD infection in the current study was 1.85 L, which were 1.62 L in the MCL production system and 5.35 L in the commercial dairy farms. This finding was in agreement with the report from the previous study (Jemberu et al., 2014) who reported an average milk loss of 1.8 L both in the MCL and pastoral production system in Ethiopia. Beyi (2012) reported a milk loss of 8.45 L per day per infected cow in the commercial dairy farms in central Ethiopia, which is higher than the loss in the current study. In the current study, milk production was reduced by 51.7% during the period of illness which was lower than 62.74 reported by Beyi (2012), 73.3% by Bayissa, Ayelet, Kyule, Jibril, and Gelaye (2011) and more than 75% by Jemberu et al. (2014) in Ethiopia. A similar reduction of 51.8% to the present finding was reported in Pakistan (Ferrari, Tasciotti, Khan, & Kiani, 2013). Elsewhere in the world, higher milk yield reduction of 66% in Bangladesh (Chowdhury et al., 1993), 65% in Kenya (Lyons *et al*., 20l5) and 62.3% in South Sudan (Barasa et al., 2008) were reported from previous works.

The herd level cost of treatment in the current study especially in MCL system was very low (USD 0.16) as only about 2.2% of infected animals were treated. The herd level cost of treatment in the commercial farms (USD 5.7) was also lower than the USD 67 per infected farm reported in central Ethiopia (Beyi, 2012). Most of the animal owners in the MCL system in the current study areas were reluctant to get their FMD‐infected animals treated and hence low treatment cost as compared with commercial farms. This is because of a traditional belief that treatment of clinically FMD‐infected animals aggravates the disease severity (locally they called this condition ‘Tila’) and leads to death.

The USD 13.34 per ox mean economic loss due to draught power loss in the MCL production system in the current study was in agreement with the report of Jemberu et al. (2014); they reported a mean loss of USD 15 per ox in North Wollo, Ethiopia. The mean effective working days an ox stay out of work (4.8 days) and the mean duration of illness in an ox (27 days) in the current study were again in agreement with the report of Jemberu et al. (2014).Whereas the mean effective working days lost was higher than a report by Mersie et al. (1992) in Eastern Ethiopia. The economic loss from an ox stay out of work is due to the renting of replacement oxen for plowing and threshing.

The farm level average economic loss incurred by FMD‐infected commercial dairy farms in Gondar town (USD 459.1) in the current study was significantly higher than the loss from MCL production system (USD 34). This is in spite of higher herd level morbidity in the MCL system. One obvious reason for this difference could be the herd size difference which, on average, was 6.7 cattle for CLM system and 17.6 cattle for commercial dairy system (Table 2) but this alone cannot be explain the large difference observed between the two systems. Other factors for the higher economic loss in the commercial dairy farms can be the high reduction of milk yield per infected cow and high proportion of milking cows in the farms. This makes FMD an economically more important problem in commercial farms as compared with traditional subsistence systems. An average farm level economic loss higher than the result of the current study was estimated previously as USD 1671.5 in commercial dairy farms in central Ethiopia (Beyi, 2012). Jemberu et al. (2014) reported mean total economic losses per herd of USD 76 for the MCL production system which is higher than the finding of the current study in the MCL production system.

During the current study in the MCL production system, the largest losses at infected animal level occur when the animal dies. Mortality losses were on average USD 100.8 per died animal in the MCL production system, which was lower than animal level mortality losses reported previously in MCL production system (USD 129) and pastoral production system (USD 151) (Jemberu et al., 2014). This difference may be due to the categories and breed of died animals which have different market values. There was no mortality in the commercial dairy farms in the current study. This may be because infected animals in the commercial dairy farm got better treatment for secondary complication and other palliative care.

In the commercial dairy farms, the largest loss at infected animal level was due to milk production loss. The USD 97.5 loss per affected lactating cow which is lower than the finding of Beyi (2012) in central Ethiopia commercial dairy farms who reported an average mean loss of USD 108.96. An average total losses of USD 86 and USD 493 per infected dairy cow were reported in Turkey from local and Holstein‐Friesian cows, respectively (Şentürk & Yalçin, 2008), in which the loss from Holstein‐Friesian cows is higher than the loss from the current study.

The economic losses due to milk loss in the current study varied from USD 1.9 to USD 200 and USD 48.6 to USD 216.7 per affected lactating cow in the MCL production system and commercial dairy farms, respectively, depending on the level of production of affected animal, severity of the disease, the severity of milk reduction and the duration of the illness. Despite the large variation within the production system, the economic impact of the disease was more severe in commercial production system.

Although FMD outbreak morbidity and mortality data was collected both from cattle and small ruminants, economic impact was estimated only in cattle as the economic loss parameters such as milk loss and draft loss were applicable only to cattle in the study areas. Other less visible economic impacts such as weight loss, delayed growth and breeding, infertility, etc., that also apply to small ruminants were not considered as they were difficult to estimate in the current study. That was the limitation of the current study and as such the estimated economic loss could considered as underestimation of the holistic economic impact of the FMD outbreak to herd owners. Other limitation of this study was that potential confounders of milk reduction such as change in stage of lactation, management factors, etc., during the outbreak period were not controlled in a counter factual design. This might affect the accuracy of milk loss estimate that can be purely ascribed to FMD.

## 5 CONCLUSIONS

The morbidity in the infected herds in cattle was higher in the MCL production system than in commercial dairy farms indicating high transmission within and between herds in the MCL production system. Serotype O was the dominant serotype circulating in the study area. The economic losses associated with the outbreaks are variable among households and are higher for commercial dairy farms than for households within the MCL production system. In the MCL production system, the largest component of the economic loss at animal level was due to mortality loss followed by milk loss and draft loss, but in commercial dairy farms the largest loss was due to milk loss. In commercial dairy farms, there was no mortality loss indicating that there was a good palliative care for infected animals. Although the current estimates of the economic losses were limited to the most visible direct loss of the disease and treatment cost, still they constitute significant loss to farmers’ income. Future research should focus on the implementation and evaluations of effective control measures that reduce the morbidity and mitigate the economic losses.

## ETHICS STATEMENT

The authors confirm that the ethical policies of the journal, as noted on the journal's author guidelines page, have been adhered to and the appropriate ethical review committee approval has been received.

## CONFLICT OF INTEREST

None.

## APPENDIX 1

Questionnaire for interviewing smallholder farmers in the MCL production system and farm owners in the commercial dairy farms to collect data for economic impact study.

I. Questionnaire for collecting data for economic impact study.

1. Name of the herd owner________________

2. Herd location: District/farm_________________

- Kebele ________________Village______________________

3. Morbidity, mortality and associated factors recording sheet for cattle per herd

**Table:**

Category	Pregnant cows		Lactating cows		Dry cows		Young stock		Calf		Draft Oxen		Total		Remark
Herd size	L	C	L	C	L	C	L	C	L	C	L	C	L	C	
No. of sick															
No. of dead															
No. of aborted cow			‐	‐‐	‐	‐	‐	‐	‐	‐	‐	‐			
No. animal treated															
Average treatment cost per affected animal (Birr)															
Cost per died animal(Birr)															

L, local breed; C, cross breed.

4. Recording sheet for drop in milk yield due to FMD

**Table:**

Herd/Farm	Breed	No of lactating cows	Duration of less production in days	Normal average daily milk yield before sickness(liter)	Average daily milk yield during sickness (liter)	Milk price per liter	Remark
							

5. How long did the affected Ox stay out of work during the illness? ____________

6. The daily renting price of an Ox______________

7. What is the daily cost of laborer in your locality? __________________

8. What is the cost of milk per litter in your locality/nearby town?_________________

II. Questioner for collecting morbidity and mortality data for Sheep and goat.

1. Do you have sheep? Yes______ No ______

2. If yes, did the FMD affect your sheep? Yes______ No______

3. How many of your sheep are affected? (Affected/total number)

3.1. For lambs (pre‐weaning) _________/ __________

3.2. For yearling_________/ ___________

3.3. For adult___________/ __________

How many of your sheep died? (Died/total number/unit price)

4.1. For lambs (pre‐weaning) _________/ __________/ ________

4.2. For yearling_________/ ___________/ _________________

4.3. For adult___________/ __________/ __________________
